# Contribution of Tumor Endothelial Cells in Cancer Progression

**DOI:** 10.3390/ijms19051272

**Published:** 2018-04-24

**Authors:** Kyoko Hida, Nako Maishi, Dorcas A. Annan, Yasuhiro Hida

**Affiliations:** 1 Vascular Biology, Frontier Research Unit, Institute for Genetic Medicine, Hokkaido University, Sapporo 060-0815, Japan; mnako@igm.hokudai.ac.jp (N.M.); annandorcasam@gmail.com (D.A.A.); 2 Department of Cardiovascular and Thoracic Surgery, Hokkaido University Graduate School of Medicine, Sapporo 060-0815, Japan; yhida@med.hokudai.ac.jp

**Keywords:** angiogenesis, antiangiogenic therapy, blood vessel, cancer, endothelial cell, tumor microenvironment, metastasis, drug resistance

## Abstract

Tumor progression depends on the process of angiogenesis, which is the formation of new blood vessels. These newly formed blood vessels supply oxygen and nutrients to the tumor, supporting its progression and providing a gateway for tumor metastasis. Tumor angiogenesis is regulated by the balance between angiogenic activators and inhibitors within the tumor microenvironment. Because the newly formed tumor blood vessels originate from preexisting normal vessels, tumor blood vessels, and tumor endothelial cells (TECs) have historically been considered to be the same as normal blood vessels and endothelial cells; however, evidence of TECs’ distinctive abnormal phenotypes has increased. In addition, it has been revealed that TECs constitute a heterogeneous population. Thus, TECs that line tumor blood vessels are important targets in cancer therapy. We have previously reported that TECs induce cancer metastasis. In this review, we describe recent studies on TEC abnormalities related to cancer progression to provide insight into new anticancer therapies.

## 1. Introduction

Tumor angiogenesis refers to the formation of new blood vessels within a tumor, which is essential for tumor progression. Tumor blood vessels supply the tumor with oxygen and nutrients, which are required for growth, in addition to removing waste products from tumor tissues and providing a gateway for tumor metastasis [1,2]. These blood vessels consist of tumor endothelial cells (TECs), which line the insides of the blood vessels, and perivascular cells (pericytes for microvessels and smooth muscle cells for arteries and veins), which surround the blood vessels externally and play a role in blood vessel contraction and relaxation. In adults, endothelial cells (ECs) are mostly quiescent and proliferate only once every 150 d; however, angiogenesis might be induced by angiogenic factors (i.e., an angiogenic “switch”), such as the vascular endothelial growth factor (VEGF), when tissues become hypoxic, as occurs in pathologies such as cancer and wounds. Among all forms of pathological angiogenesis, tumor angiogenesis is the most important. Once an angiogenic switch is turned on, cancer begins to grow and metastasize; however, without angiogenesis, cancer cannot grow beyond a few millimeters, which would not be threatening to human life. In fact, autopsies have reported that dormant, small millimeter-sized mammary carcinomas were detected in 40% of patients who died of a different disease [3].

The concept that cancer depends on angiogenesis and that angiogenesis inhibition can have anticancer results was first proposed in 1971 by Folkman [1]. Folkman’s initial concept was not easily accepted; however, basic research has since proved that it was correct. Evidence has shown the presence of angiogenic molecules, especially following VEGF cloning [4,5]. Furthermore, anti-VEGF antibody was reported to have antiangiogenic and antitumor effects [6]. More than a decade has passed since the first antiangiogenic drug, bevacizumab, was approved in 2004. The bases for pursuing this therapy are as follows: (1) the survival of a large population of tumor cells depends on a few TECs, such that targeting these TECs might be more efficient than targeting the tumor cells; (2) because TECs exhibit similar characteristics regardless of their tumor of origin, a single, effective antiangiogenic drug could be used to treat many forms of cancer; and (3) it was believed that TECs in cancer stroma are genetically stable, unlike tumor cells; therefore, they do not become drug resistant.

Thus, antiangiogenic drugs that mainly target the VEGF/VEGF receptor signaling pathway have been administered in combination with chemotherapeutic drugs in many types of cancers; however, although antiangiogenic drugs were believed to be less toxic than other cytotoxic drugs, recent studies have shown that they might also induce severe side effects, such as lethal hemoptysis [7,8] and intestinal perforations [9,10]. Accordingly, an important goal in cancer therapy is to develop new and safer tumor antiangiogenic agents, which will depend on a thorough understanding of the biology of TECs.

In this review, we describe recent studies on TEC abnormalities related to cancer progression to provide insights into new anticancer therapies.

## 2. Molecules that Regulate Angiogenesis

Vascular endothelial growth factor: VEGF (VEGF-A), which is induced by hypoxia, is the most well-known angiogenic factor. In cancer cells, the activation of oncogenes and the mutation of tumor suppressor genes also cause VEGF upregulation. VEGF activates ECs through paracrine signaling and stimulates cell migration and the proliferation of ECs, resulting in the induction of angiogenesis. VEGF also enhances vascular permeability [4]. VEGFR-1, VEGFR-2, and VEGFR-3 are tyrosine kinase VEGF receptors. VEGF receptor (R)-1 and VEGFR-2 are expressed in blood ECs, whereas VEGFR-3 is expressed in lymphatic ECs. VEGFR-2 is the most important receptor in angiogenesis signaling. VEGFR-1 is also expressed in monocytes and macrophages and is involved in angiogenesis by stimulating the mobilization of these cells from the bone marrow. Soluble VEGFR-1, which is spliced from VEGFR-1 and has a higher affinity than VEGFR-2, interferes with VEGF–VEGFR-2 binding by trapping VEGF [11]. In addition to VEGF, cancer cells secrete other angiogenic factors, such as basic fibroblast growth factor (bFGF), angiopoietins (Ang), hepatocyte growth factor, epidermal growth factor (EGF), platelet-derived growth factor (PDGF), and placental-derived growth factor. Ang-1 directly induces adhesion between endothelial cells, possibly resulting in mural cell adhesion to ECs by processing the maturation of blood vessels. Ang-2, an Ang secreted mainly from ECs, activates Tie-2, which is much weaker than Ang-1 and acts as an antagonist of Ang-1 to detach pericytes from ECs. PDGF-BB, which is also secreted from ECs, is also important for angiogenesis and has two receptors—α-receptor and β-receptor. PDGF-BB acts on the PDGFR-β that is expressed in the pericytes and attracts the pericytes to the newly formed blood vessels; however, to initiate angiogenesis, the pericytes must detach from ECs in the normal and stable blood vessels.

**Figure 1:**
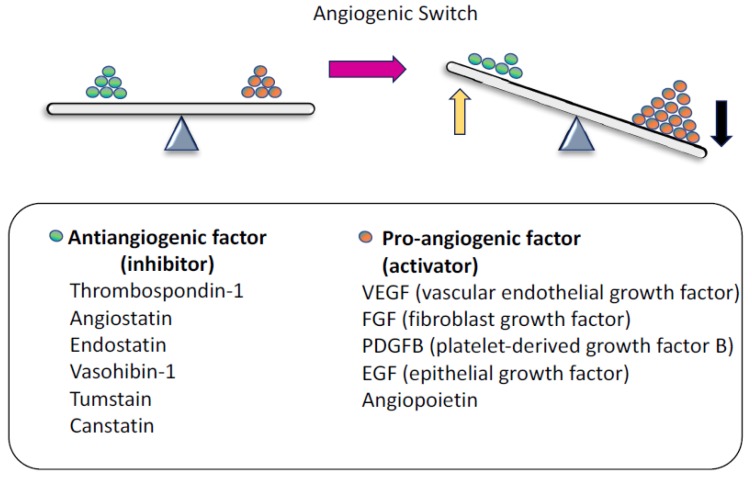
Angiogenesis is regulated by a balance of angiogenic and antiangiogenic factors. In angiogenesis, Pro-angiogenic supporters activate endothelial cells (ECs). In contrast, antiangiogenic factors suppress EC activation. When the angiogenic switch is turned on, there are more angiogenic (black arrow) than antiangiogenic factors present (yellow arrow).

Several angiogenic inhibitor genes have also been identified, such as thrombospondin-1 (TSP-1), Notch ligand Delta-like 4 (DLL4), vasohibin-1 (VASH1), and Down syndrome critical region-1 (DSCR-1). TSP-1 expression is regulated by tumor suppressor p53. In various types of cancer, p53 is mutated and causes the downregulation of TSP-1 [12]. DLL4, VASH1, and DSCR-1 are expressed in ECs and act as angiogenesis inhibitors. DLL4, which is secreted from cells located at the tip of the blood vessel branch, or “tip” cells, regulates vessel sprouting by binding the Notch1 receptor in stalk cells [13]. VASH1, which is expressed in ECs, locates in the termination zone [14] in angiogenesis and terminates the process. DSCR-1 was identified as a calcineurin inhibitor upstream of the nuclear factor of activated T-cells and is activated by VEGF [15]. In addition, there are endogenous angiogenic inhibitors that are cleaved from molecules that are not directly related to angiogenesis. For example, angiostatin is produced by plasminogen cleavage [2], and endostatin [16] and tumstatin [17] are protein fragments cleaved from basement membrane collagen types XVIII and IV, respectively. Collagen type XVIII gene is coded on chromosome 21 in humans. It is known that patients with Down syndrome have fewer incidences of cancer because of their elevated blood endostatin levels, possibly the result of the additional copy of chromosome 21 in their DNA [18]. Angiogenesis is regulated in a complex manner by the balance of these angiogenic activators and inhibitors (Figure 1). A balance between these factors is required for the physiological regulation of angiogenesis. In pathological angiogenesis, such as tumor angiogenesis, the balance between angiogenic and antiangiogenic factors is not equal. In this case, angiogenic activator levels become higher than angiogenic suppressor levels. Underexposure of either of these factors causes ECs to be stimulated and to respond.

**Figure 2:**
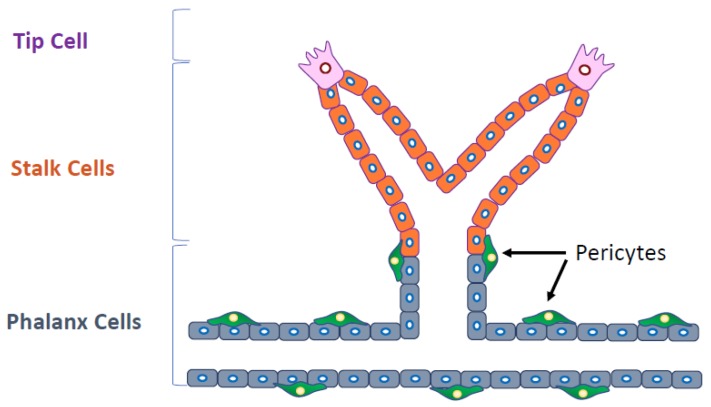
Several types of endothelial cells (ECs) are involved in angiogenesis. Cells at the tip of the blood vessel branch (tip cells) guide the direction of vessel sprouting. Stalk cells, which are highly proliferative, follow tip cells, and phalanx cells improve the perfusion and oxygenation of newly formed blood vessels. Pericytes attach to phalanx cells (black arrows).

Several types of ECs are involved in angiogenesis. Tip cells guide the direction of vessel sprouting. Stalk cells, which are highly proliferative, follow tip cells, and phalanx cells improve the perfusion and oxygenation of newly formed blood vessels. Molecular signaling pathways are differentially activated in these cells (Figure 2). Thus, it has been recognized that the specific signaling pathways and the specific ECs involved should be the target of antiangiogenic therapy. Furthermore, pericytes or smooth muscle cells stabilize the blood vessels, finalizing the angiogenesis process.

## 3. Tumor Endothelial Cells

Phenotypic differences at the molecular and functional levels have been identified using TECs and normal ECs (NECs) isolated from tumor and normal tissues, respectively; however, for many years, most studies on tumor angiogenesis have been conducted using NECs, such as normal human umbilical vein ECs (HUVECs), given that isolation of TECs has been difficult because ECs are usually enmeshed in complex tissue, and only a small fraction of the cells within these tissues are ECs. In addition to the technical difficulties, there might have been concerns about trials to isolate TECs themselves because they were at times considered to lose their specific phenotype soon after being isolated from tumor tissue.

In 2000, St. Croix et al. succeeded in isolating ECs from colon carcinoma and normal colonic mucosa and compared the gene expression profiles between TECs and NECs in a relatively low number of uncultured cells using serial analysis of gene expression. They identified the specific genes for TEC and designated them as tumor endothelial markers (TEMs) [19] and reported that TEM8 could be a target of antiangiogenic therapy [20].

Since then, there have been several studies to elucidate the molecular differences between TECs and NECs using global analysis [21,22]. In some studies, vascular cells have been captured by laser-captured microdissection to identify vascular makers. The authors have described that these markers might not be strictly specific to TECs because laser-capture microdissection-captured cells contain not only ECs but also mural cells, such as pericytes or smooth muscle cells; however, this approach is also important in identifying TEC-specific markers, especially when using human clinical specimens. In addition, TECs have been compared with ECs in the tissue under physiological angiogenesis in an attempt to identify molecules that are specific to TECs, not ECs, in physiologic angiogenesis. For example, Seaman et al. [23] compared TECs and ECs in angiogenic corpus luteum and identified several TEC markers (TEMs), including CD276, which is known to be a regulator of T cell-mediated immune response [24]. Van Beijnum et al. [25] identified TEC-specific molecules, including high mobility group box 1 protein (HMGB1), by comparing gene profiles between TECs and placental ECs. In most of these global analyses described above, TECs were not cultured and their biological phenotype remains unclear.

Contrary to these, there have been studies based on cultured TECs. It was demonstrated that TECs isolated from human renal cell carcinoma did not undergo senescence, unlike NECs, and were resistant to apoptotic stimuli with enhanced Akt activation and decreased expression of the tumor suppressor phosphatase and tensin homolog deleted from chromosome 10 (PTEN) [26]. We have also purified TECs in an attempt to better understand their TEC.

Because possible contamination by tumor cells has been a concern for TEC culture, we eliminated the human tumor cells in a subculture of mouse TECs isolated from a human tumor xenografted into mice using diphtheria toxin (DT) [27]. DT was used because the heparin-binding EGF (HB-EGF), which is expressed in human but not in mouse cells, is a DT receptor; consequently, DT is toxic to HB-EGF-expressing human cells but not to mouse cells [28]. Using purified TECs, we found that they retain their previously reported properties, such as TEM gene expression and less apoptotic features, even in culture [29] and demonstrated that TEC-specific molecules, such as C-X-C chemokine receptor type 7 (CXCR7) and LOX [30,31], include differences in their responsiveness to growth factors [32,33], such as VEGF and EGF and their proangiogenic phenotypes [29,34]. It has been demonstrated that TECs secrete several factors that enhance their survival in an autocrine manner [26,33,35,36].

## 4. Drug Resistance and Cytogenetic Abnormalities in Tumor Endothelial Cells

Several cytogenetic abnormalities, such as aneuploidy and abnormal centrosomes, have been reported in TECs from mouse tumors [27] and human renal carcinomas [37]. These TECs were characterized by structural aberrations, such as nonreciprocal translocations, missing chromosomes, marker chromosomes, and double minutes using multiple-colored fluorescent in situ hybridization analysis. Individual TECs had different cytogenetic profiles, which indicated that they were heterogeneous and not clonal. Thus, TECs have hallmarks of chromosomal instability. Studies have confirmed that these abnormalities were not a result of tumor cell contaminants. A recent study has demonstrated that TECs and circulating TECs showed aneuploidy [38] and that they can also have abnormal centrosomes. The normal function of centrosomes is to establish cell polarity and to properly segregate the chromosomes. Defects in centrosome function with loss of polarity and with chromosome missegregation have been detected in aggressive human malignant tumors. We found that TECs have between one and five centrosomes and thus mimic tumor cells. Because TECs continue to proliferate in culture, it appears that these cells, like tumor cells, lack the normal cell cycle checkpoints that inhibit mitosis in response to centrosome abnormalities [27].

On the other hand, there have been several reports that tumor cells, such as glioblastoma or lymphoma cells, transdifferentiate into TECs, a process that causes them to contain cytogenetic abnormalities [39,40]. TECs have long been considered normal diploid cells that, unlike tumor cells, do not mutate and or develop drug resistance; however, aneuploid TECs might have different properties. Some antiangiogenic drugs have been shown to lose their effectiveness over time possibly a result of acquired resistance of the target cells.

Cytogenetic abnormalities indicate genetic instability and most likely explain the frequency observed in TEC resistance to chemotherapeutic agents, such as renal carcinoma-derived TEC resistance to vincristine [26], hepatocellular carcinoma-derived TEC resistance to 5-fluorouracil and adriamycin [41,42], and tumor-derived VEGF-mediated TEC resistance to paclitaxel with ATP-binding Cassette Sub-family B Member 1 (ABCB1) upregulation [42].

Tumors in which the expression of MDR1/p-glycoprotein (P-gp) is upregulated are resistant to paclitaxel [42]. Higher levels of MDR1 mRNA were detected in metastatic tumor-derived TECs than in nonmetastatic tumor-derived TECs. Complex abnormal karyotypes and excessive aneuploidy are associated with cancer cells containing multidrug-resistant genes [43]. Similarly, we have reported that metastatic tumor-derived TECs have a more complex abnormal karyotype than nonmetastatic tumor-derived TECs [44]. These cytogenetic abnormalities could contribute to drug resistance in high metastatic tumor-derived TECs. The molecules that are expressed in drug-resistant TECs can be important therapeutic targets for overcoming resistance to anti-angiogenic therapy. We have found that P-gp inhibitor verapamil resensitizes TECs to paclitaxel, leading to antitumor effects [45].

## 5. Heterogeneity of Tumor Endothelial Cells

ECs are morphologically and functionally heterogeneous. For example, the rolling velocity and arrest frequency of leukocytes at NEC junctions are different from those in the central areas [46]. Interorgan differences in ECs have also been reported [47]. In preexisting blood vessels, stem-like ECs with a proangiogenic phenotype have been identified, and ECs that express ABCB1/P-gp have been reported in residential normal and tumor blood vessels [48,49]. In addition, P-gp and endothelial barrier antigens are heterogeneously expressed in rat-brain blood vessels, particularly at the single-cell level, suggesting the heterogeneous formation of the blood–brain barrier [50].

In addition, there are many examples of TEC heterogeneity [27]. We have demonstrated that some TECs show upregulated expressions of the stem cell marker aldehyde dehydrogenase (ALDH). These ALDH^high^ TECs are more proangiogenic and drug resistant, with a higher grade of chromosomal abnormality, than ALDH^low^ TECs [51]. Within the tumor vasculature, the morphology and pericyte coverage of tumor blood vessels vary depending on the type of tumor and progression stage [47,52,53]. In addition, we have reported that these heterogeneities [44] are dependent on tumor malignancy. The blood vessels of metastatic tumors are more immature with fewer pericytes than those of nonmetastatic tumors [44]. These features could be attributed to the higher hypoxic nature of metastatic tumors compared with that of nonmetastatic tumors. TECs isolated from metastatic tumors demonstrated a more proangiogenic phenotype than those isolated from nonmetastatic tumors, with the upregulation of several angiogenesis-related genes, such as VEGFR-1, VEGFR-2, and VEGF [35,54]. During tumor neovascularization, TECs use matrix metalloproteinases (MMPs) to breach the basement membrane and degrade the extracellular matrix, thus allowing TEC migration into the tumor during angiogenesis. TECs from metastatic tumors also showed higher invasive potential than TECs from nonmetastatic tumors with the upregulation of gelatinase/collagenase IV MMPs (MMP-2 and MMP-9). These TEC characteristics support tumor progression and metastasis [44].

TECs might also acquire heterogeneity during cancer therapy. For example, it has been reported that Ang-2 expression is upregulated during anti-VEGF therapy. This might be a mechanism by which to escape this therapy. Indeed, dual inhibition of Ang-2 and VEGF receptor signaling prolonged survival in glioblastoma [55]. Anti-VEGF therapy transiently normalizes immature tumor vessel structure and improves vessel function; therefore, drug delivery is improved and radiotherapy efficacy is better from oxygenation of the tumor tissue where blood perfusion is induced [56]. On the other hand, sustained antiangiogenic therapy eventually leads to an ischemic change in the tumor and worsens hypoxia, which results in tumor malignancy [57]. Thus, we must develop biomarkers of the vessel normalization time frame to design the optimal scheduling protocols for combination therapies. Unfortunately, there are no reliable predictors or biomarkers for identifying the vascular normalization time frame during antiangiogenic therapy, although a recent study has reported that apelin is upregulated as a biomarker for the vessel normalization time frame during antiangiogenic therapy [58].

## 6. Mechanisms of Tumor Endothelial Cell Abnormality

**Figure 3:**
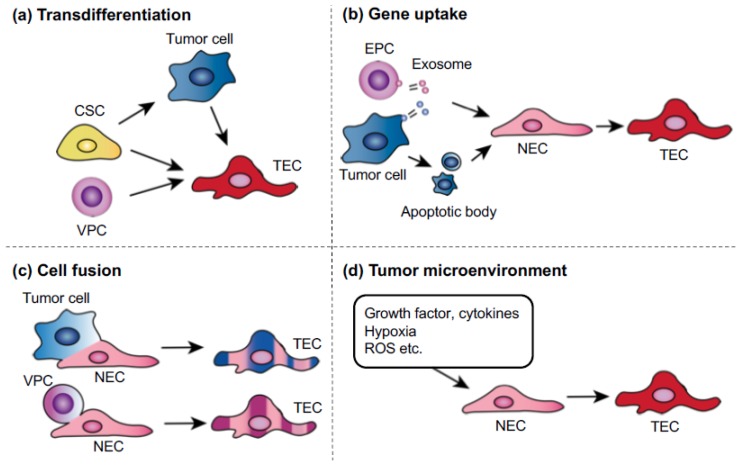
Possible mechanisms of tumor endothelial cells (TEC) abnormalities. (**a**) Transdifferentiation: tumor cells, cancer stem cells, or vascular progenitor cells (VPCs) might transdifferentiate into TECs. (**b**) Uptake of oncogenes or gene transfer: ECs can take up human tumor oncogenes by phagocytosis of apoptotic bodies or exosomes, which are released from either endothelial progenitor cells or tumor cells. (**c**) Cell fusion: malignant tumor cells can fuse with normal endothelial cells (NECs) or circulating VPCs. (**d**) Tumor microenvironment: growth factors or cytokines in the tumor microenvironment might be factors that cause genetic instability. Hypoxia in tumors is known to cause genetic changes, such as the upregulation of survival factors, not only in tumor cells.

Several mechanisms have been suggested as possible causes of TEC abnormality (Figure 3). Interactions between tumor and stromal cells have been reported as a mechanism by which stromal cells become abnormal structures, such as tumor-associated macrophages and cancer-associated fibroblasts, causing tumor progression and metastasis. In our study, NECs cocultured in a metastatic tumor cell-conditioned medium showed upregulated proangiogenic gene expression and changes that resembled some TEC phenotypes [44], suggesting that tumor-secreted factors influence TECs. Hypoxia might also cause TEC abnormalities. The immature and leaky tumor blood vessels cause high tissue pressure within the tumor, leading to the collapse of the blood vessels and resulting in hypoxia [59,60]. Many studies have demonstrated that hypoxia is closely related to cancer malignancy. It induces excessive VEGF production and vascular permeability, which might cause TEC abnormalities. The increased vascular permeability compromises the blood flow in tumor blood vessels, which further decreases oxygen and nutrient supply, causing physiological stress on the tumor. The persisting hypoxia, together with the secretion of cytokines such as VEGF, promotes tumor revascularization by inducing the mobilization of bone marrow-derived endothelial progenitor cells toward the cancer [61]. Furthermore, hypoxia might cause genetic instability in ECs. An excess of VEGF induces abnormal centrosome structures in HUVECs [62]. We have also shown that hypoxia-induced reactive oxygen species causes aneuploidy in NECs and human microvascular ECs [63] and that TEC abnormalities might also be attributed to the origin of these cells. It was reported that cancer stem cell glioblastomas differentiated into TECs [40,64], although a recent study showed that glioblastoma cells give rise to pericytes rather than to ECs [65]. The transdifferentiation of lymphoma cells into TECs was also proposed to underlie TEC abnormality and heterogeneity [39]. This transdifferentiation might be a mechanism by which TEC chromosomes become abnormal in some, but not all, tumor models. For example, we did not observe that TECs transdifferentiated from tumor cells in studies using a cross-species model (human tumor and mouse ECs) in which it was possible to show that ECs were not derived from human tumor cells using species-specific fluorescent in situ hybridization probes or antibodies [37]. The full range of mechanisms that account for TEC abnormalities must be determined in future studies.

## 7. Tumor Endothelial Cells’ Roles in Cancer Progression

Morphologically abnormal tumor vasculature leads to tumor cell intravasation during tumor metastasis. VEGF signaling loosens the tight junctions between ECs. For example, VE-cadherin, which makes up the EC–EC junctions, is internalized after VEGF stimulation [66], which induces vascular permeability. The immature structure of tumor blood vessels that lack smooth muscles and pericytes also leads to tumor cell transendothelial migration [67]. In addition to this passive entry route, TECs might also actively promote tumor cell metastasis, the reason being that it has been reported that TECs secrete cytokines called “angiocrine factors” such as interleukin-6, VEGF-A, and bFGF [68]. For example, FGF4 secreted from B-cell lymphoma cells activates FGFR1 in TECs and upregulates the Notch ligand Jagged-1 in ECs. In turn, Jagged-1 in ECs reciprocally induces Notch2–Hey1 in lymphoma cells [69], which makes tumor cells more invasive and chemoresistant [70]. Other studies have also demonstrated that the Notch signals in ECs are important in cancer stem cells [71] and promote neutrophil infiltration [72].

**Figure 4:**
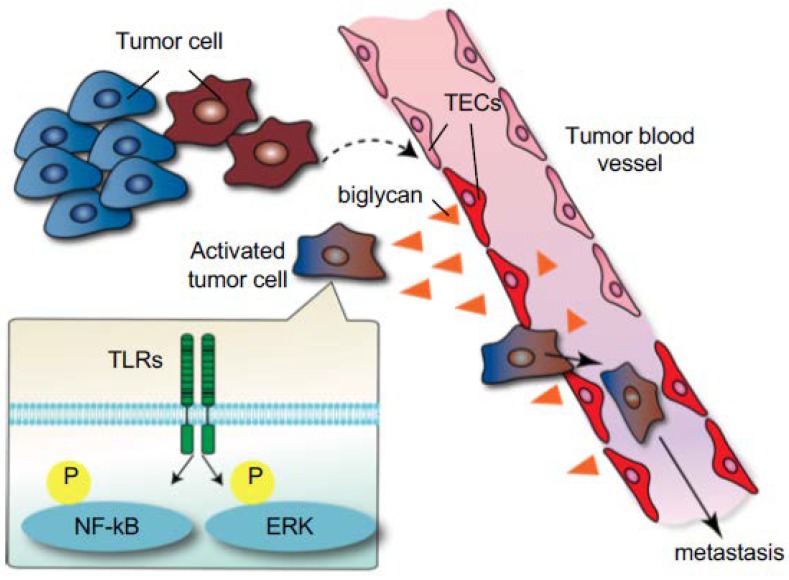
Induction of tumor metastasis by tumor endothelial cells TECs through biglycan secretion. The interaction between tumor cells and the microenvironment alters TEC phenotypes (dotted arrow). In turn, these altered TECs express high levels of biglycan, which induces tumor cells to metastasize through the activation of NF-κB and ERK signaling (black arrow) [73]. (Reprinted from Maishi et al., *Sci. Rep.* **2016**, with permission from *Sci. Rep.*).

We have reported the role of TECs in the initial steps of tumor metastasis [73]. Because TECs in metastatic tumors express higher levels of angiocrine factors than those in nonmetastatic tumors [44], it was speculated that TECs might also affect tumor cell behavior. In vitro data revealed that TECs from metastatic tumors attract and adhere to tumor cells to a greater extent than TECs from nonmetastatic tumors or NECs. In addition, tumor cell transendothelial migration was observed on the monolayer formed by TECs from metastatic tumors. We found that biglycan, a small leucine-rich repeat proteoglycan, was one of the molecules responsible for these phenotypes in metastatic tumor TECs. Indeed, TEC biglycan facilitated the migration of toll-like receptor-expressing tumor cells through the activation of nuclear factor-kappa B (NF-κB) and extracellular signal-regulated kinase (ERK) signaling. The biglycan that was secreted from TECs increased the number of circulating tumor cells and lung metastases in vivo. Biglycan levels in the plasma of patients with cancer were higher than those in healthy volunteers, particularly in metastatic cases. These results suggested that TECs provide this key molecule to tumor cells for hematogenous metastasis (Figure 4). Furthermore, the biglycan promoter was markedly demethylated in TECs from metastatic tumors, but not in other ECs, and this demethylation shows that epigenetic dysregulation might be one of the mechanisms involved in TEC abnormalities [73]. Collectively, altered TECs facilitate cancer progression and metastasis in the tumor microenvironment.

## 8. Conclusions

TECs differ from NECs both morphologically and physiologically. Contrary to previous presumptions, TECs are not homogeneous, and they are affected by the complex tumor microenvironment. In addition, there is a bidirectional interaction between TECs and tumor cells, through which TECs actively affect tumor cells and play a role in cancer progression. Elucidation of TEC biology with the help of additional studies would provide a new target for anticancer therapy and diagnostics.

## References

[1] Folkman J. (1971). Tumor angiogenesis: Therapeutic implications. N. Engl. J. Med..

[2] Folkman J., Kerbel R. (2002). Role of angiogenesis in tumor growth and metastasis. Clinical translation of angiogenesis inhibitors. Semin. Oncol..

[3] Nielsen M., Thomsen J.L., Primdahl S., Dyreborg U., Andersen J.A. (1987). Breast cancer and atypia among young and middle-aged women: A study of 110 medicolegal autopsies. Br. J. Cancer.

[4] Senger D.R., Galli S.J., Dvorak A.M., Perruzzi C.A., Harvey V.S., Dvorak H.F. (1983). Tumor cells secrete a vascular permeability factor that promotes accumulation of ascites fluid. Science.

[5] Ferrara N., Henzel W.J. (1989). Pituitary follicular cells secrete a novel heparin-binding growth factor specific for vascular endothelial cells. Biochem. Biophys. Res. Commun..

[6] Kim K.J., Li B., Winer J., Armanini M., Gillett N., Phillips H.S., Ferrara N. (1993). Inhibition of vascular endothelial growth factor-induced angiogenesis suppresses tumour growth in vivo. Nature.

[7] Johnson D.H., Fehrenbacher L., Novotny W.F., Herbst R.S., Nemunaitis J.J., Jablons D.M., Langer C.J., DeVore R.F., Gaudreault J., Damico L.A. (2004). Randomized phase II trial comparing bevacizumab plus carboplatin and paclitaxel with carboplatin and paclitaxel alone in previously untreated locally advanced or metastatic non-small-cell lung cancer. J. Clin. Oncol..

[8] Keedy V.L., Sandler A.B. (2007). Inhibition of angiogenesis in the treatment of non-small cell lung cancer. Cancer Sci..

[9] Kindler H.L., Friberg G., Singh D.A., Locker G., Nattam S., Kozloff M., Taber D.A., Karrison T., Dachman A., Stadler W.M. (2005). Phase II trial of bevacizumab plus gemcitabine in patients with advanced pancreatic cancer. J. Clin. Oncol..

[10] Saif M.W., Elfiky A., Salem R.R. (2007). Gastrointestinal perforation due to bevacizumab in colorectal cancer. Ann. Surg. Oncol..

[11] Shibuya M., Claesson-Welsh L. (2006). Signal transduction by VEGF receptors in regulation of angiogenesis and lymphangiogenesis. Exp. Cell Res..

[12] Dameron K.M., Volpert O.V., Tainsky M.A., Bouck N. (1994). Control of angiogenesis in fibroblasts by p53 regulation of thrombospondin-1. Science.

[13] Noguera-Troise I., Daly C., Papadopoulos N.J., Coetzee S., Boland P., Gale N.W., Lin H.C., Yancopoulos G.D., Thurston G. (2006). Blockade of Dll4 inhibits tumour growth by promoting non-productive angiogenesis. Nature.

[14] Sato Y. (2010). The Vasohibin Family. Pharmaceuticals.

[15] Minami T., Horiuchi K., Miura M., Abid M.R., Takabe W., Noguchi N., Kohro T., Ge X., Aburatani H., Hamakubo T. (2004). Vascular endothelial growth factor- and thrombin-induced termination factor, Down syndrome critical region-1, attenuates endothelial cell proliferation and angiogenesis. J. Biol. Chem..

[16] Folkman J. (2006). Antiangiogenesis in cancer therapy—Endostatin and its mechanisms of action. Exp. Cell Res..

[17] Maeshima Y., Manfredi M., Reimer C., Holthaus K.A., Hopfer H., Chandamuri B.R., Kharbanda S., Kalluri R. (2001). Identification of the anti-angiogenic site within vascular basement membrane-derived tumstatin. J. Biol. Chem..

[18] Ryeom S., Folkman J. (2009). Role of endogenous angiogenesis inhibitors in Down syndrome. J. Craniofac. Surg..

[19] St Croix B., Rago C., Velculescu V., Traverso G., Romans K.E., Montgomery E., Lal A., Riggins G.J., Lengauer C., Vogelstein B. (2000). Genes expressed in human tumor endothelium. Science.

[20] Nanda A., St Croix B. (2004). Tumor endothelial markers: New targets for cancer therapy. Curr. Opin. Oncol..

[21] Lu C., Bonome T., Li Y., Kamat A.A., Han L.Y., Schmandt R., Coleman R.L., Gershenson D.M., Jaffe R.B., Birrer M.J. (2007). Gene Alterations Identified by Expression Profiling in Tumor-Associated Endothelial Cells from Invasive Ovarian Carcinoma. Cancer Res..

[22] Buckanovich R.J., Sasaroli D., O’Brien-Jenkins A., Botbyl J., Hammond R., Katsaros D., Sandaltzopoulos R., Liotta L.A., Gimotty P.A., Coukos G. (2007). Tumor vascular proteins as biomarkers in ovarian cancer. J. Clin. Oncol..

[23] Van Beijnum J.R. (2006). Gene expression of tumor angiogenesis dissected: Specific targeting of colon cancer angiogenic vasculature. Blood.

[24] Seaman S., Stevens J., Yang M.Y., Logsdon D., Graff-Cherry C., St Croix B. (2007). Genes that Distinguish Physiological and Pathological Angiogenesis. Cancer Cell.

[25] Van Beijnum J.R., Nowak-Sliwinska P., van den Boezem E., Hautvast P., Buurman W.A., Griffioen A.W. (2013). Tumor angiogenesis is enforced by autocrine regulation of high-mobility group box 1. Oncogene.

[26] Bussolati B., Deambrosis I., Russo S., Deregibus M.C., Camussi G. (2003). Altered angiogenesis and survival in human tumor-derived endothelial cells. FASEB J..

[27] Hida K., Hida Y., Amin D.N., Flint A.F., Panigrahy D., Morton C.C., Klagsbrun M. (2004). Tumor-associated endothelial cells with cytogenetic abnormalities. Cancer Res..

[28] Arbiser J.L., Raab G., Rohan R.M., Paul S., Hirschi K., Flynn E., Price E.R., Fisher D.E., Cohen C., Klagsbrun M. (1999). Isolation of mouse stromal cells associated with a human tumor using differential diphtheria toxin sensitivity. Am. J. Pathol..

[29] Matsuda K., Ohga N., Hida Y., Muraki C., Tsuchiya K., Kurosu T., Akino T., Shih S.-C., Totsuka Y., Klagsbrun M. (2010). Isolated tumor endothelial cells maintain specific character during long-term culture. Biochem. Biophys. Res. Commun..

[30] Maishi N., Ohga N., Hida Y., Akiyama K., Kitayama K., Osawa T., Onodera Y., Shinohara N., Nonomura K., Shindoh M. (2012). CXCR7: A novel tumor endothelial marker in renal cell carcinoma. Pathol. Int..

[31] Osawa T., Ohga N., Akiyama K., Hida Y., Kitayama K., Kawamoto T., Yamamoto K., Maishi N., Kondoh M., Onodera Y. (2013). Lysyl oxidase secreted by tumour endothelial cells promotes angiogenesis and metastasis. Br. J. Cancer.

[32] Amin D.N., Hida K., Bielenberg D.R., Klagsbrun M. (2006). Tumor endothelial cells express epidermal growth factor receptor (EGFR) but not ErbB3 and are responsive to EGF and to EGFR kinase inhibitors. Cancer Res..

[33] Tsuchiya K., Hida K., Hida Y., Muraki C., Ohga N., Akino T., Kondo T., Miseki T., Nakagawa K., Shindoh M. (2010). Adrenomedullin antagonist suppresses tumor formation in renal cell carcinoma through inhibitory effects on tumor endothelial cells and endothelial progenitor mobilization. Int. J. Oncol..

[34] Kurosu T., Ohga N., Hida Y., Maishi N., Akiyama K., Kakuguchi W., Kuroshima T., Kondo M., Akino T., Totsuka Y. (2011). HuR keeps an angiogenic switch on by stabilising mRNA of VEGF and COX-2 in tumour endothelium. Br. J. Cancer.

[35] Bussolati B., Assenzio B., Deregibus M.C., Camussi G. (2006). The proangiogenic phenotype of human tumor-derived endothelial cells depends on thrombospondin-1 downregulation via phosphatidylinositol 3-kinase/Akt pathway. J. Mol. Med..

[36] Muraki C., Ohga N., Hida Y., Nishihara H., Kato Y., Tsuchiya K., Matsuda K., Totsuka Y., Shindoh M., HIDA K. (2011). Cyclooxygenase-2 inhibition causes antiangiogenic effects on tumor endothelial and vascular progenitor cells. Int. J. Cancer.

[37] Akino T., Hida K., Hida Y., Tsuchiya K., Freedman D., Muraki C., Ohga N., Matsuda K., Akiyama K., Harabayashi T. (2009). Cytogenetic abnormalities of tumor-associated endothelial cells in human malignant tumors. Am. J. Pathol..

[38] Lin P.P., Gires O., Wang D.D., Li L., Wang H. (2017). Comprehensive in situ co-detection of aneuploid circulating endothelial and tumor cells. Sci. Rep..

[39] Streubel B., Chott A., Huber D., Exner M., Jager U., Wagner O., Schwarzinger I. (2004). Lymphoma-specific genetic aberrations in microvascular endothelial cells in B-cell lymphomas. N. Engl. J. Med..

[40] Wang R., Chadalavada K., Wilshire J., Kowalik U., Hovinga K.E., Geber A., Fligelman B., Leversha M., Brennan C., Tabar V. (2010). Glioblastoma stem-like cells give rise to tumour endothelium. Nature.

[41] Xiong Y.Q., Sun H.C., Zhang W., Zhu X.D., Zhuang P.Y., Zhang J.B., Wang L., Wu W.Z., Qin L.X., Tang Z.Y. (2009). Human Hepatocellular Carcinoma Tumor-derived Endothelial Cells Manifest Increased Angiogenesis Capability and Drug Resistance Compared with Normal Endothelial Cells. Clin. Cancer Res..

[42] Akiyama K., Ohga N., Hida Y., Kawamoto T., Sadamoto Y., Ishikawa S., Maishi N., Akino T., Kondoh M., Matsuda A. (2012). Tumor Endothelial Cells Acquire Drug Resistance by MDR1 Up-Regulation via VEGF Signaling in Tumor Microenvironment. Am. J. Pathol..

[43] Duensing S., Munger K. (2002). Human papillomaviruses and centrosome duplication errors: Modeling the origins of genomic instability. Oncogene.

[44] Ohga N., Ishikawa S., Maishi N., Akiyama K., Hida Y., Kawamoto T., Sadamoto Y., Osawa T., Yamamoto K., Kondoh M. (2012). Heterogeneity of tumor endothelial cells: Comparison between tumor endothelial cells isolated from high- and low-metastatic tumors. Am. J. Pathol..

[45] Akiyama K., Maishi N., Ohga N., Hida Y., Ohba Y., Alam M.T., Kawamoto T., Ohmura H., Yamada K., Torii C. (2015). Inhibition of Multidrug Transporter in Tumor Endothelial Cells Enhances Antiangiogenic Effects of Low-Dose Metronomic Paclitaxel. Am. J. Pathol..

[46] Mundhekar A.N., Bullard D.C., Kucik D.F. (2006). Intracellular heterogeneity in adhesiveness of endothelium affects early steps in leukocyte adhesion. Am. J. Physiol. Cell Physiol..

[47] Molema G. (2010). Heterogeneity in endothelial responsiveness to cytokines, molecular causes, and pharmacological consequences. Semin. Thromb. Hemost..

[48] Naito H., Kidoya H., Sakimoto S., Wakabayashi T., Takakura N. (2011). Identification and characterization of a resident vascular stem/progenitor cell population in preexisting blood vessels. EMBO J..

[49] Naito H., Wakabayashi T., Kidoya H., Muramatsu F., Takara K., Eino D., Yamane K., Iba T., Takakura N. (2016). Endothelial Side Population Cells Contribute to Tumor Angiogenesis and Antiangiogenic Drug Resistance. Cancer Res..

[50] Saubamea B., Cochois-Guegan V., Cisternino S., Scherrmann J.M. (2012). Heterogeneity in the rat brain vasculature revealed by quantitative confocal analysis of endothelial barrier antigen and P-glycoprotein expression. J. Cereb. Blood Flow Metab..

[51] Ohmura-Kakutani H., Akiyama K., Maishi N., Ohga N., Hida Y., Kawamoto T., Iida J., Shindoh M., Tsuchiya K., Shinohara N. (2014). Identification of tumor endothelial cells with high aldehyde dehydrogenase activity and a highly angiogenic phenotype. PLoS ONE.

[52] Langenkamp E., Molema G. (2009). Microvascular endothelial cell heterogeneity: General concepts and pharmacological consequences for anti-angiogenic therapy of cancer. Cell Tissue Res..

[53] Nagy J.A., Dvorak H.F. (2012). Heterogeneity of the tumor vasculature: The need for new tumor blood vessel type-specific targets. Clin. Exp. Metast..

[54] Adya R., Tan B.K., Punn A., Chen J., Randeva H.S. (2008). Visfatin induces human endothelial VEGF and MMP-2/9 production via MAPK and PI3K/Akt signalling pathways: Novel insights into visfatin-induced angiogenesis. Cardiovasc. Res..

[55] Peterson T.E., Kirkpatrick N.D., Huang Y., Farrar C.T., Marijt K.A., Kloepper J., Datta M., Amoozgar Z., Seano G., Jung K. (2016). Dual inhibition of Ang-2 and VEGF receptors normalizes tumor vasculature and prolongs survival in glioblastoma by altering macrophages. Proc. Natl. Acad. Sci. USA.

[56] Jain R.K. (2001). Normalizing tumor vasculature with anti-angiogenic therapy: A new paradigm for combination therapy. Nat. Med..

[57] Winkler F., Kozin S.V., Tong R.T., Chae S.S., Booth M.F., Garkavtsev I., Xu L., Hicklin D.J., Fukumura D., di Tomaso E. (2004). Kinetics of vascular normalization by VEGFR2 blockade governs brain tumor response to radiation: Role of oxygenation, angiopoietin-1, and matrix metalloproteinases. Cancer Cell.

[58] Zhang L., Takara K., Yamakawa D., Kidoya H., Takakura N. (2015). Apelin as a marker for monitoring the tumor vessel normalization window during antiangiogenic therapy. Cancer Sci..

[59] Sato Y. (2011). Persistent vascular normalization as an alternative goal of anti-angiogenic cancer therapy. Cancer Sci..

[60] Helfrich I., Scheffrahn I., Bartling S., Weis J., von Felbert V., Middleton M., Kato M., Ergun S., Schadendorf D. (2010). Resistance to antiangiogenic therapy is directed by vascular phenotype, vessel stabilization, and maturation in malignant melanoma. J. Exp. Med..

[61] Gao D., Nolan D., McDonnell K., Vahdat L., Benezra R., Altorki N., Mittal V. (2009). Bone marrow-derived endothelial progenitor cells contribute to the angiogenic switch in tumor growth and metastatic progression. Biochim. Biophys. Acta.

[62] Taylor S.M., Nevis K.R., Park H.L., Rogers G.C., Rogers S.L., Cook J.G., Bautch V.L. (2010). Angiogenic factor signaling regulates centrosome duplication in endothelial cells of developing blood vessels. Blood.

[63] Kondoh M., Ohga N., Akiyama K., Hida Y., Maishi N., Towfik A.M., Inoue N., Shindoh M., Hida K. (2013). Hypoxia-induced reactive oxygen species cause chromosomal abnormalities in endothelial cells in the tumor microenvironment. PLoS ONE.

[64] Ricci-Vitiani L., Pallini R., Biffoni M., Todaro M., Invernici G., Cenci T., Maira G., Parati E.A., Stassi G., Larocca L.M. (2010). Tumour vascularization via endothelial differentiation of glioblastoma stem-like cells. Nature.

[65] Cheng L., Huang Z., Zhou W., Wu Q., Donnola S., Liu J.K., Fang X., Sloan A.E., Mao Y., Lathia J.D. (2013). Glioblastoma stem cells generate vascular pericytes to support vessel function and tumor growth. Cell.

[66] Gavard J., Gutkind J.S. (2006). VEGF controls endothelial-cell permeability by promoting the beta-arrestin-dependent endocytosis of VE-cadherin. Nat. Cell Biol..

[67] Chang Y.S., di Tomaso E., McDonald D.M., Jones R., Jain R.K., Munn L.L. (2000). Mosaic blood vessels in tumors: Frequency of cancer cells in contact with flowing blood. Proc. Natl. Acad. Sci. USA.

[68] Butler J.M., Kobayashi H., Rafii S. (2010). Instructive role of the vascular niche in promoting tumour growth and tissue repair by angiocrine factors. Nat. Rev. Cancer.

[69] Cao Z., Ding B.S., Guo P., Lee S.B., Butler J.M., Casey S.C., Simons M., Tam W., Felsher D.W., Shido K. (2014). Angiocrine factors deployed by tumor vascular niche induce B cell lymphoma invasiveness and chemoresistance. Cancer Cell.

[70] Cao Z., Scandura J.M., Inghirami G.G., Shido K., Ding B.S., Rafii S. (2017). Molecular Checkpoint Decisions Made by Subverted Vascular Niche Transform Indolent Tumor Cells into Chemoresistant Cancer Stem Cells. Cancer Cell.

[71] Zhu T.S., Costello M.A., Talsma C.E., Flack C.G., Crowley J.G., Hamm L.L., He X., Hervey-Jumper S.L., Heth J.A., Muraszko K.M. (2011). Endothelial cells create a stem cell niche in glioblastoma by providing NOTCH ligands that nurture self-renewal of cancer stem-like cells. Cancer Res..

[72] Wieland E., Rodriguez-Vita J., Liebler S.S., Mogler C., Moll I., Herberich S.E., Espinet E., Herpel E., Menuchin A., Chang-Claude J. (2017). Endothelial Notch1 Activity Facilitates Metastasis. Cancer Cell.

[73] Maishi N., Ohba Y., Akiyama K., Ohga N., Hamada J., Nagao-Kitamoto H., Alam M.T., Yamamoto K., Kawamoto T., Inoue N. (2016). Tumour endothelial cells in high metastatic tumours promote metastasis via epigenetic dysregulation of biglycan. Sci. Rep..

